# 41. Assessing Past vs Present COVID-19 Infection: A Survey of Criteria for Discontinuing Precautions in Asymptomatic Patients

**DOI:** 10.1093/ofid/ofab466.041

**Published:** 2021-12-04

**Authors:** Shruti K Gohil, Annabelle De St Maurice, Deborah S Yokoe, Deborah S Yokoe, Stuart H Cohen, Francesca J Torriani, Jonathan Grein, Philip A Robinson, Shannon C Mabalot, Paula Pedrani, Jessica Park, Richard Platt, Susan S Huang

**Affiliations:** 1 UC Irvine School of Medicine, IRVINE, California; 2 University of California, Los Angeles, Los Angeles, CA; 3 University of California, San Francisco, San Francisco, CA; 4 University of California, Davis, Sacramento, CA; 5 University of California, San Diego, San Diego, CA; 6 Cedars-Sinai Medical Center, Los Angeles, CA; 7 Hoag Hospital, Irvine, California; 8 Sharp Memorial Hospital, San Diego, CA; 9 University of California, Irvine, Irvine, California; 10 Harvard Medical School, Boston, Massachusetts

## Abstract

**Background:**

COVID-19 patients can remain positive by PCR-testing for several months. Pre-admission or pre-procedure testing can identify recovered asymptomatic patients who may no longer be contagious but would require precautions according to current CDC recommendations (10 days). This can result in unintended consequences, including procedure delays or transfer to appropriate care (e.g., psychiatric or post-trauma patients requiring admission to COVID-19 units instead of psychiatric or rehabilitation facilities, respectively).

**Methods:**

We conducted a structured survey of healthcare epidemiologists and infection prevention experts from the SHEA Research Network between March-April, 2021. The 14-question survey, presented a series of COVID-19 PCR+ asymptomatic patient case scenarios and asked respondents if (1) they would consider the case recovered and not infectious, (2) if they have cleared precautions in such cases, and if so, (3) how many transmission events occurred after discontinuing precautions. The survey used one or a combination of 5 criteria: history of COVID-19 symptoms, history of exposure to a household member with COVID-19, COVID-19 PCR cycle threshold (CT), and IgG serology. Percentages were calculated among respondents for each question.

**Results:**

Among 60 respondents, 56 (93%) were physicians, 51 (86%) were hospital epidemiologists, and 46 (77%) had >10y infection prevention experience. They represented facilities that cumulatively cared for >29,000 COVID-19 cases; 46 (77%) were academic, and 42 (69%) were large ( >400 beds). One-third to one-half would consider an incidentally found PCR+ case as recovered based on solo criteria, particularly those with two consecutive high CTs or COVID IgG positivity recovered (53-55%) (Table 1). When combining two criteria, half to four-fifths of respondents deemed PCR+ cases to be recovered (Table 2). Half of those had used those criteria to clear precautions (45-64%) and few to none experienced a subsequent transmission event resulting from clearance.

**Conclusion:**

The majority of healthcare epidemiologists consider a combination of clinical and diagnostic criteria as recovered and many have used these to clear precautions without high numbers of transmission.

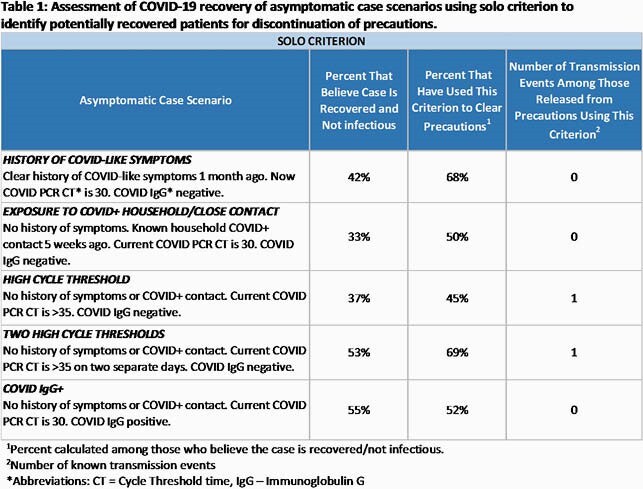

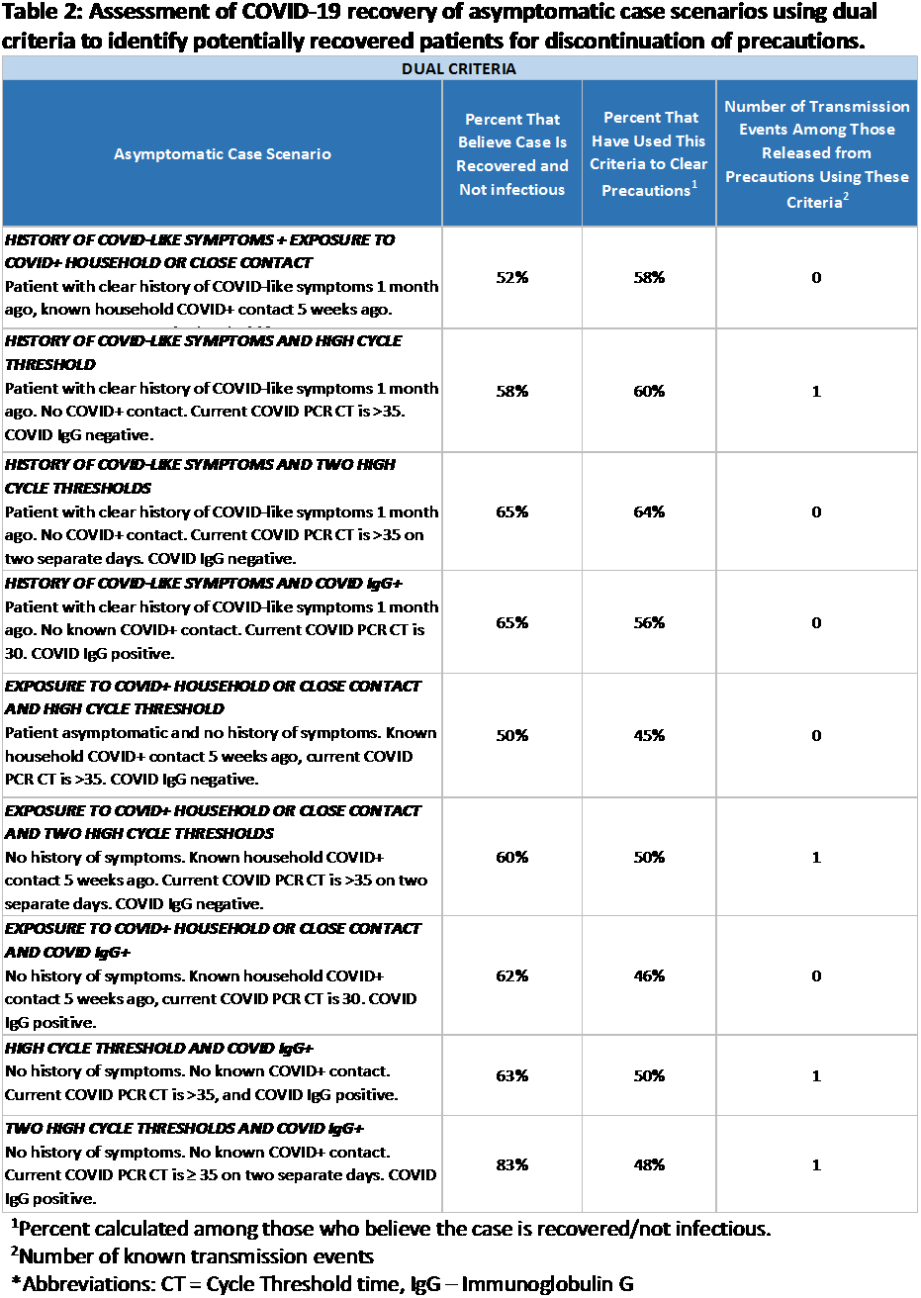

**Disclosures:**

**Shruti K. Gohil, MD, MPH**, **Medline** (Other Financial or Material Support, Co-Investigator in studies in which participating hospitals and nursing homes received contributed antiseptic and cleaning products)**Molnycke** (Other Financial or Material Support, Co-Investigator in studies in which participating hospitals and nursing homes received contributed antiseptic and cleaning products)**Stryker (Sage**) (Other Financial or Material Support, Co-Investigator in studies in which participating hospitals and nursing homes received contributed antiseptic and cleaning products) **Deborah S. Yokoe, MD, MPH**, Nothing to disclose **Stuart H. Cohen, MD**, **Seres** (Research Grant or Support) **Jonathan Grein, MD**, **Gilead** (Other Financial or Material Support, Speakers fees) **Richard Platt, MD, MSc**, **Medline** (Research Grant or Support, Other Financial or Material Support, Conducted studies in which participating hospitals received contributed antiseptic product)**Molnlycke** (Other Financial or Material Support, Conducted studies in which participating hospitals received contributed antiseptic product) **Susan S. Huang, MD, MPH**, **Medline** (Other Financial or Material Support, Conducted studies in which participating hospitals and nursing homes received contributed antiseptic and cleaning products)**Molnlycke** (Other Financial or Material Support, Conducted studies in which participating hospitals and nursing homes received contributed antiseptic and cleaning products)**Stryker (Sage**) (Other Financial or Material Support, Conducted studies in which participating hospitals and nursing homes received contributed antiseptic and cleaning products)**Xttrium** (Other Financial or Material Support, Conducted studies in which participating hospitals and nursing homes received contributed antiseptic and cleaning products)

